# Facilitating plasmid nuclear delivery by interfering with the selective nuclear pore barrier

**DOI:** 10.1002/btm2.10136

**Published:** 2019-06-22

**Authors:** Ihab Azzam, Ivan Liashkovich, Isabelle Luchtefeld, Ivan U. Kouzel, Victor Shahin

**Affiliations:** ^1^ Institute of Physiology II University of Münster Münster Germany; ^2^ Institute of Hygiene University of Münster Münster Germany

**Keywords:** nanomedicine, nanoparticles, nuclear pores, pharmacology, physiology

## Abstract

Nuclear pore complexes (NPCs) are sophisticated transporters assembled from diverse proteins termed nucleoporins (Nups). They control all nucleocytoplasmic transport and form a stringent barrier between the cytosol and the nucleus. While selective receptor‐mediated transport enables translocation of macromolecules up to striking sizes approaching megadalton‐scale, the upper cutoff for diffusion is at 40 kDa. Raising the cutoff is of particular importance for nuclear delivery of therapeutic nanoparticles, for example, gene and chemotherapy. In this work, we set out to present compounds capable of raising the cutoff to an extent enabling nuclear delivery of 6 kbp pDNA (150 kDa) in cultured human vascular endothelial cells. Of all tested compounds one is singled out, 1,6‐hexanediol (1,6‐HD). Our observations reveal that 1,6‐HD facilitates nuclear delivery of pDNA in up to 10–20% of the tested cells, compared to no delivery at all in control conditions. It acts by interfering with bonds between Nups that occupy the NPC channel and confer transport selectivity. It also largely maintains cell viability even at high concentrations. We envisage that 1,6‐HD may serve as a lead substance and usher in the design of potent new strategies to increase nuclear delivery of therapeutic nanoparticles.

## 1 INTRODUCTION

Nuclear pore complexes (NPCs) are proteinaceous structures that span the nuclear envelope at regular distances and form the sole pathways for nucleocytoplasmic transport. An individual NPC consists of multiple copies of ~30 different nucleoporins (Nups), which fulfill different functions.^1^ Two thirds of the Nups are engaged in building the stable NPC scaffold and its anchorage to the nuclear envelope among others. The remaining third is made up of highly flexible and dynamic brush‐like Nups with motifs rich in hydrophobic phenylalanine and glycine (FG) repeats, connected through hydrophilic spacer sequences.^2^ FG‐Nups are abundant inside the center of the NPC channel and are responsible for selective transport.^2^ Their structural arrangement inside the NPC channel generates a barrier that determines the upper cut‐off for passive diffusion of molecules, which is at a diameter of 5 nm or a size of 40 kDa.^1^ Receptor‐mediated transport on the other hand, is highly selective and enables the translocation of far greater molecules up to 39 nm.^3^ Such molecules, for instance proteins, bear nuclear localization signals, which are recognized by soluble transport receptors. The latter regularly shuttle between the cytosol and the nucleus and are naturally capable of interacting with FG‐Nups inside the NPC channel thereby mediating cargo translocation through the barrier.^4^ The exact structural configuration of FG‐Nups which confers the selective barrier function remains debatable and diverse models have been postulated.^5^ The “selective phase model”^6^ relies on the assumption that cohesive FG‐Nups intrinsically tend to be attracted to one another. The resulting interchangeable bonds form a saturated homogeneous hydrogel‐like mesh that serves as a selective sieve. Transport factors bearing their specific cargos dissolve in the FG‐hydrogel and translocate within milliseconds while unselective molecules are kept out owing to the mesh size in the hydrogel. The “forest model”^7^ suggests a fixed structural arrangement with two separate transport routes for small and large molecules, the former along the NPC scaffold while the latter through the channel, respectively. The “virtual gating^8^/polymer brush model^9^” proposes that noncohesive FG‐Nups do not attract but repel each other, thereby acting much like polymer brushes. Owing to their highly dynamic conformation, the unfolded brushes form an entropic barrier at the NPC channel, which repels unspecific molecules. Receptor‐bound cargos, however, bind to the brushes, lead to their folding, lower the entropic barrier, and eventually translocate through the channel. Increasing the upper cut‐off limit of the FG‐Nups barrier is highly beneficial for delivering diverse therapeutic nanoparticles to their place of action, the nucleus, for instance for cancer and gene therapies.^10^, ^11^ Several amphiphilic alcohols disrupt NPC selectivity and therefore break its barrier function by acting on FG‐Nups, for example, trans‐1,2‐cyclohexanediol (1,2‐TCHD).^12^, ^13^, ^14^, ^15^ Upon the addition of this NPC barrier breaker (NBB), NPCs become permeable to 70 kDa dextran^16^, ^17^ and single pDNA particles.^13^ 1,2‐TCHD, however, exhibits significant cytotoxic effects.^13^, ^16^ In this study, we present 1,6‐hexanediol as a potent NBB, that facilitates nuclear delivery of pDNA with significantly higher efficacy as compared to 1,2‐TCHD, while maintaining viability of cells.

## 2 MATERIALS AND METHODS

### 2.1 Confocal fluorescence microscopy for nuclear barrier permeability measurements

EA.hy926 cells were cultured as described in our previous work.^18^ NBBs were diluted to specified concentrations, and added 30 min prior to permeabilization of cells for 5 min with digitonin solution (20 μg/mL) in transport buffer (TB, 20 mM HEPES, 110 mM K‐acetate, 5 mM Na‐acetate, 2 mM Mg‐acetate, 1 mM EGTA [pH 7.3], 2 mM DTT). Then, the TB was replaced with digitonin‐free buffer containing rhodamine‐labeled pDNA. Images were performed at the mid‐plane of the cell nuclei using Leica SP8 confocal laser scanning microscope equipped with hybrid detection system for photon counting (Leica, Wetzlar, Germany), at a rate of one image per minute for 30 min. Nuclear influx dynamics of the fluorescent molecules was analyzed by measuring the ratio between the intranuclear fluorescence intensity and the extracellular background intensity. Nuclear import of pDNA was measured at 24 hr post‐transfection. For the first 6 hr, the cells remained in UltraCruz transfection medium containing 1 μg pDNA and for the latter 18 hr this medium was replaced with fresh, antibiotics‐free medium which contained the NBBs at concentrations of 0, 2, and 4%. The cells remained in this medium during imaging. To assess nuclear import of fluorescent pDNA, the intranuclear fluorescence intensity was compared to the cytosolic intensity and the ratio was calculated.

### 2.2 Plasmid handling and transfection

A GFP coding sequence (ggggatccaccggtcgcc) was subcloned into vector backbone containing NLS (agcttcgaattcATG) using the Infusion HD Cloning Kit™ (by Clontech/Takara Bio, Saint‐Germain‐en‐Laye, France). The plasmid pEGFP N1 Lifeact was used as template DNA for the PCR beforehand. Labeling of pDNA with rhodamine was carried out using the Label IT® Nucleic Acid Labeling Kit (by Mirus, Göttingen, Germany). For transfection experiments cells were seeded at a concentration of 2 × 10^5^ in 1 mL of antibiotics‐free standard growth medium in four well plates. The cells were left for 24 hr to grow to 40–80% confluence. Two solutions were prepared: the first with 1 μg of pDNA (plasmid DNA) in 50 μL plasmid transfection medium (by Santa Cruz Biotechnology, Heidelberg, Germany) and the second with 1 μL UltraCruz® transfection reagent in 50 μL plasmid transfection medium. Both solutions were incubated at room temperature for 5 min. Solution 1 was pipetted drop‐wise into Solution 2, vortexed and incubated at room temperature for 20 min. The growth medium in the plate was replaced with fresh medium and the solution mixture was pipetted into it. The plates were swirled gently and left to incubate for 24–72 hr. The medium was replaced after the initial 24 hr of incubation.

### 2.3 Treatment with NBBs

Six hours post‐transfection, the growth medium was replaced with a growth medium containing 2 and 4% of myo‐inositol (MI) or 1,6‐HD. This medium was replaced with fresh medium after 18 hr.

### 2.4 Viability assay

Viability of cells was tested with propidium iodide (PI) assay according to the manufacturer's instructions (ThermoFisher Scientific, Waltham, MA). Cells were treated with 1 μL Hoechst solution (nuclear staining, ThermoFisher Scientific) and 1 μL PI, incubated for 1 hr at 37°C, then imaged with fluorescence microscopy.

### 2.5 Western blot

Western blot was applied, according to a detailed method in our previous publication,^16^ to check for potential dissociation of FG‐Nups following treatment with NBBs. Five hundred microliters of the desired NBBs (1,6‐HD, MI, or 1,2‐TCHD) was added at the desired concentration (2% and/or 4% in TB). A 0% control was prepared with 500 μL of TB. The lysates of the EA.hy96 cells were created by adding triton‐lysis buffer (1% Triton X‐100, 150 mM NaCl, 5 mM EDTA, and 50 mM Tris–HCL) and protease inhibitor (cOmplete Mini, Roche Diagnostics GmbH, Mannheim, Germany) to the cells. The protein concentration was detected by the BCA Pierce® Protein Assay Kit (Thermo Fisher Scientific Inc.). Lysate were size‐fractionated with 10% SDS‐Page and blotted onto nitrocellulose membrane (Hybond C‐Extra; Amersham Bioscience, Amersham, United Kingdom). The membrane was blocked in 5% skim milk powder solved in transport buffer (10 mM Tris/HCL, 1,5 M NaCl) with 0.05% Tween 20. The proteins were marked by incubation of the membrane in primary antibodies (Mab414, Covance, 1:1000; Lamin A/C, Cell Signaling Technology, 1:2000; Anti‐β‐Actin, Sigma, 1:10000; Anti‐GAPDH antibody, abcam, 1:5000) added in blocking buffer overnight. And then incubated with the secondary antibodies (Goat anti mouse, DIANOVA, 1:10000; Anti‐rabbit IgG, Sigma, 1:3000) rarefied in milk for 1 hr. By using enzyme substrates (Super Signal West pico/femto, Thermo Scientific), protein bands were viewed by the gel documentation system (ChemiDoc™XRS, Bio‐Rad). For quantification, the program ImageJ was used.

### 2.6 Statistical analysis

All experimental conditions were repeated at least four times. Data are presented as mean values ± standard error of the mean (*SEM*). Results are considered as statistically significant at the probability level *p* < .05. The exact numbers are provided in the corresponding places. Statistical tests and graph production were performed using software Origin Pro 9.

## 3 RESULTS

### 3.1 Selection of NBBs

**Figure 1:**
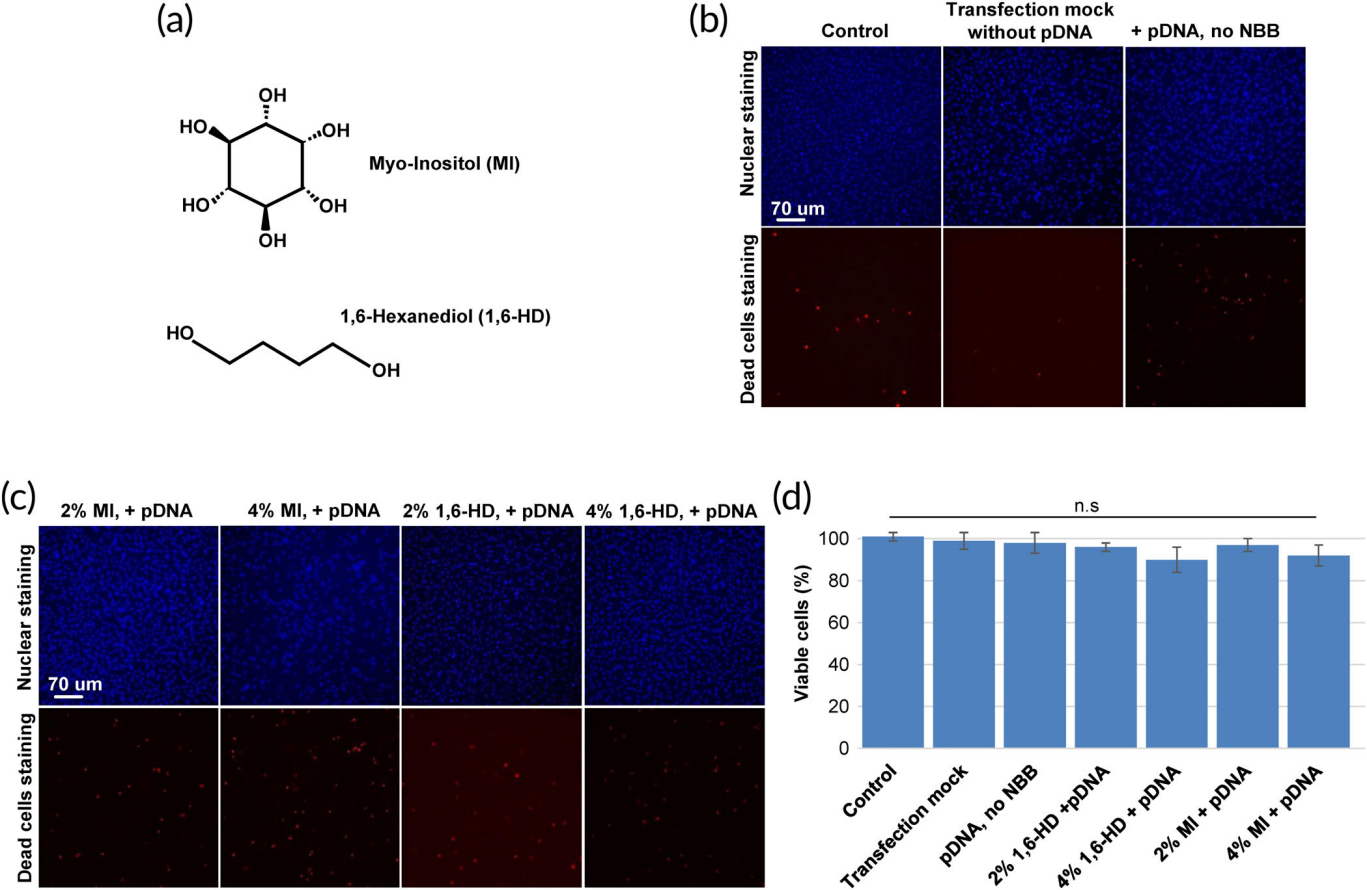
Viability assays of EA.hy926 cells for transfection experiments with pDNA, in the absence and the presence of nuclear pores barrier breakers (NBBs). Experiments were performed with the propidium iodide (PI) assay and nuclei were stained with Hoechst (blue); PI is unable to pass through plasma membranes of intact cells but can freely move through damaged membranes of dead cells, which is how it is utilized to stain them (red). (a) Chemical structures of the NBBs MI (myo‐inositol) and 1,6‐HD. (b) Viability under transfection controls without NBBs. (c) Viability in transfection experiments in presence of NBBs. (d) Summary of the viability results. Each experimental condition was carried out five times. No significant statistical differences (n.s.) compared to control (analysis of variance test)

Our previous work using 1,2‐TCHD as NBB on isolated nuclei of *Xenopus l*. ooctyes showed that it renders NPCs highly permeable to 70 kDa when applied at concentrations of 5%,^16^ by dissociating barrier‐forming FG‐Nups 62 and 98 from NPCs. 1,2‐TCHD effects were massive and irreversible, which resulted in high cytotoxicity.^16^ In this study, we set out to introduce potent yet less toxic NBBs using 1,2‐TCHD as a lead substance. Diverse compounds sharing much of the basic chemical structure were tested. Eventually, two compounds were singled out, which maintained viability of cells analyzed with PI‐based assays (Figure 1).

The first NBB 1,6‐HD (Figure 1a) is almost the linear version of 1,2‐TCHD. It exhibits insignificant cytotoxic effects (cell viability at 2% 1,6‐HD is 96 ± 2%, Mean ± *SEM*; at 4% 90 ± 6%, Figure 1d). The second NBB is MI, a poly‐alcohol with six hydroxyl groups (Figure 1a) and insignificant cytotoxicity (cell viability at 2% is 97 ± 3%, Mean ± *SEM*; at 4% 92 ± 5%, Figure 1d). It is a naturally occurring substance found in many plants and animals, where it plays diverse biological roles in cellular processes such as signal transduction, stress response, cell wall biogenesis, growth regulation, osmo‐tolerance, and membrane trafficking.^19^

### 3.2 Nuclear uptake of plasmid DNA

**Figure 2:**
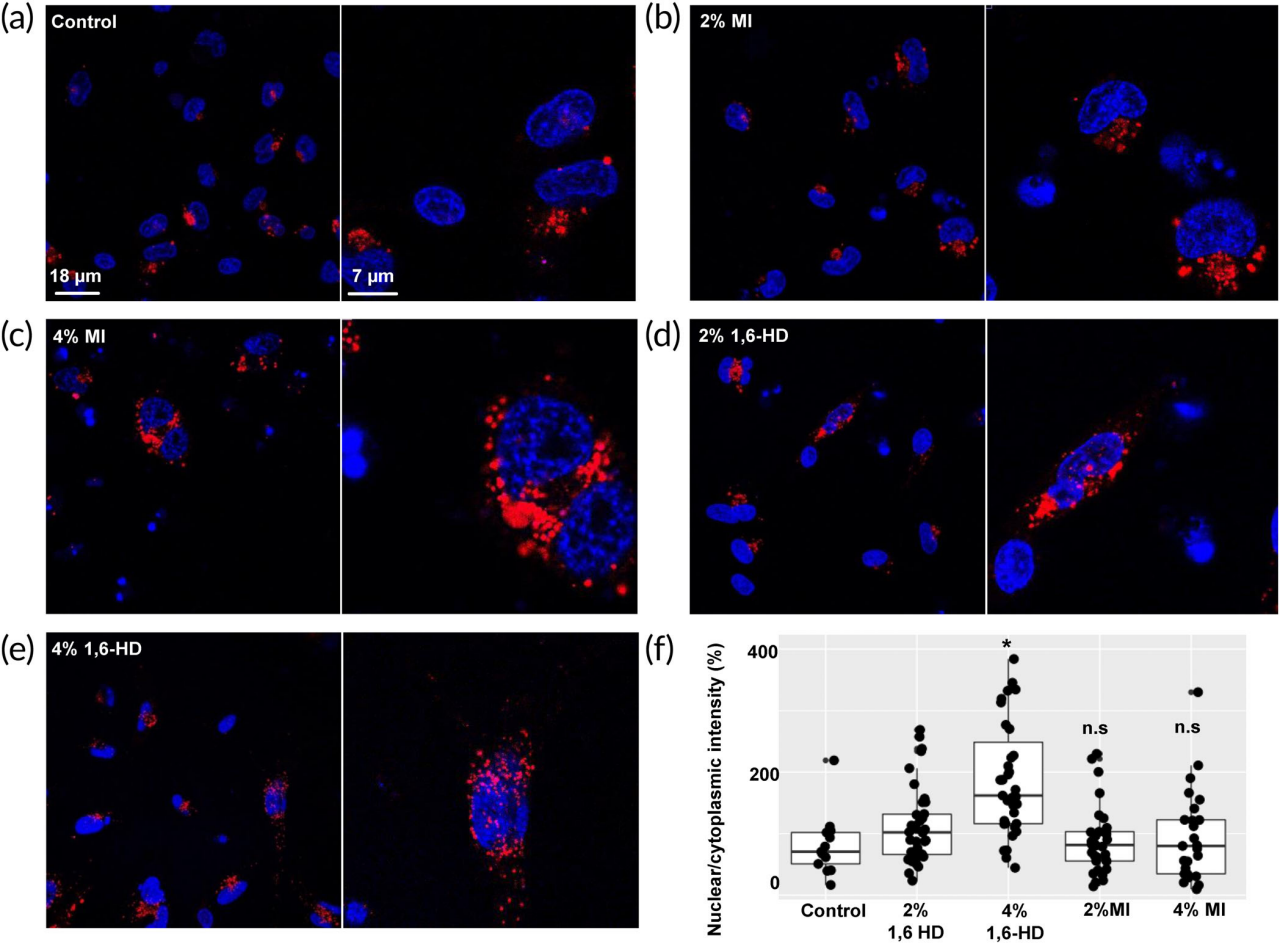
Effects of the nuclear pore barrier breakers (NBBs) myo‐inositol (MI) and 1,6‐Hexanediol (1,6‐HD) on nuclear delivery of rhodamine‐labeled pDNA (red) in EA.hy926 cells. Images are arranged each as experimental condition (left) and magnification (right): (a) control, (b) 2% MI, (c) 4% MI, (d) 2% 1,6‐HD and (e) 4% 1,6‐HD. (f) Quantification of NBBs effects on nuclear delivery of pDNA as compared to control (no NBB). Data are presented as boxplots (the central rectangle corresponds to the interquartile range (IQR), the horizontal line is the median and “whiskers” are 1.5xIQR). Nuclei were stained with Hoechst (blue). Each experimental condition was carried out five times. In each condition, at least 400 cells were analyzed. 1,6‐HD facilitates nuclear delivery of pDNA whereas MI fails to do so: at 2%, 1,6‐HD enables nuclear delivery of individual pDNA particles, not considered statistically significant (n.s., *p* > .05, pairwise comparisons using Wilcoxon rank‐sum test), while at 4% the effect is statistically significant (*, *p* < 0.001)

After chemical delivery of rhodamine‐labeled pDNA (red) into the cytosol of EA.hy926 cells, it was tracked using confocal microscopy before and after treatment with 2 and 4% MI or 1,6‐HD (Figure 2). No pDNA was detected in control nuclei conditions nor after treatment with MI. Treatment with 1,6‐HD, however, led to nuclear delivery of individual pDNA particles generally in 10–20% of the imaged cells. Nuclear delivery at 4% 1,6‐HD was statistically highly significant (*p* < .05, nonparametric test, Mann–Whitney) compared to control. At 2% 1,6‐HD there was certainly some nuclear delivery of pDNA when compared to control and 2 and 4% MI, albeit not considered statistically significant (*p* > .05). However, nuclear delivery analysis of the applied ~6 kbp pDNA cannot be dealt with exactly the same way as with standard fluorescently labeled nuclear pore permeability markers such as FITC (fluorescein isothiocyanate)‐dextrans, that are generally far smaller in size. In fact, for therapeutic nanoparticles acting inside the nucleus such as pDNA, a single molecule may be enough to ensure the desired activity as discussed previously in pharmacological contexts.^13^ In other words, the nuclear delivery data should not be looked at merely from statistical aspects but from pharmacological too.

### 3.3 Effects of the applied NBBs on FG‐Nups in NPCs

**Figure 3:**
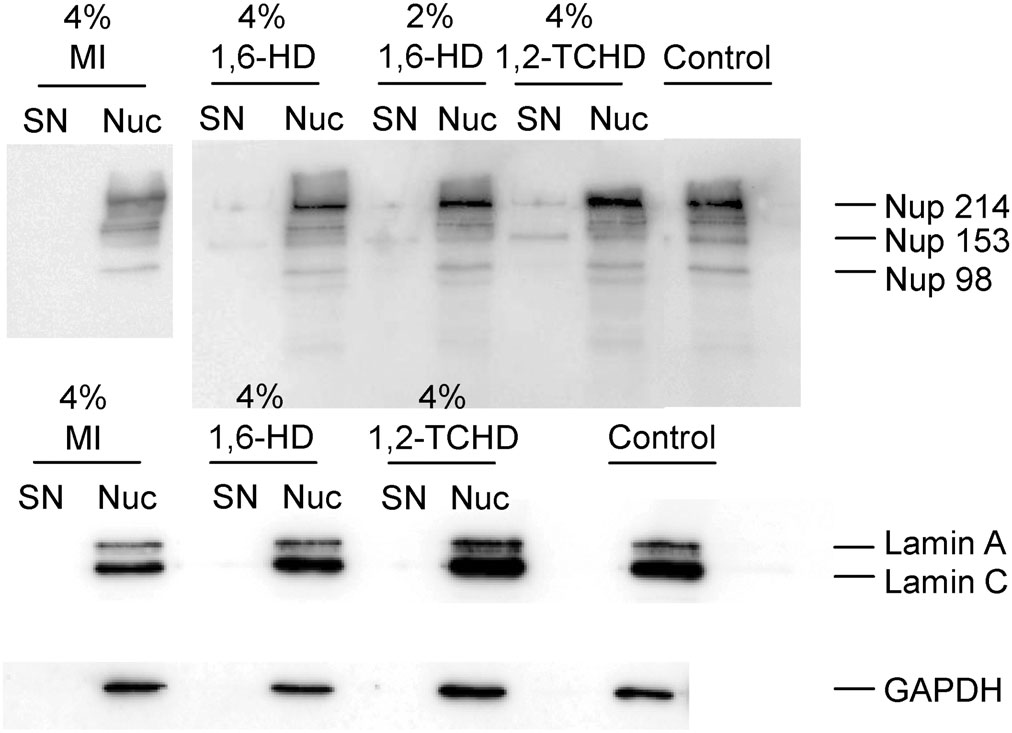
Western blots of EA.hy926 cell nuclei in absence and presence of the NBBs trans‐1,2‐cyclohexanediol (1,2‐TCHD), myo‐inositol (MI), and 1,6‐hexanediol (1,6‐HD), to study the effect of NBBs on FG‐Nups in nuclear pores. Lamin A/C and GAPDH serve as housekeeping genes controls and FG‐Nups are detected by using the antibody mAb414. 1,2‐TCHD (4%) acts as a positive control for FG‐Nups dissociation as seen in the supernatant (SN, extranclear medium) sample. In contrast to 1,2‐TCHD, neither MI nor 1,6‐HD lead to dissociation of FG‐Nups from nuclear pores. Nuc, nucleus. Western blots were repeated five times each

Several studies including ours demonstrated that aliphatic alcohols, in particular 1,2‐TCHD, may break down the nuclear barrier when applied at high concentrations, by severe disruption of the interactions between FG‐Nups.^12^, ^14^, ^15^, ^16^ This study utilizes other NBBs. Cells were permeabilized with digitonin, which permeabilizes the plasma membrane but leaves the nuclear envelope intact.^20^ They were then treated with the different NBBs to find out, using western blot analysis, whether or not the addition of the NBBs would lead to FG‐Nups dissociation from NPCs^16^, ^17^ (Figure 3). Western blots were performed with cell nuclei. In sample from control nuclei (no NBBs), there is no dissociation of FG‐Nups (lack of lanes in supernatant [SN] = extranuclear medium). In samples from 4% 1,2‐TCHD experiments, the dissociation of FG‐Nups we previously observed with this chemical,^16^ can be seen in SN. In contrast, no dissociation of FG‐Nups is observed for 1,6‐HD and MI when used at the same concentration as 1,2‐TCHD.

## 4 DISCUSSION

Vandenbroucke et al tested whether 1,2‐TCHD could facilitate nuclear uptake of pDNA in human cell lines, A549 and Vero cells.^13^ They found out that single pDNA particles translocated to the nucleus of A549 cells and discussed that the single pDNA particles may be enough to exert gene therapeutic action. However, they also pointed out the significant cytotoxicity of 1,2‐TCHD for Vero cells at concentrations starting from 1%. Our previous works reveal cytotoxicity of 1,2‐TCHD with increasing concentrations in different cell types.^16^, ^17^ Here, we present 1,6‐HD as promising lead substance for the design of pharmacologically potent NBBs facilitating nuclear delivery of nanoparticles. 1,6‐HD is well characterized and tested for toxicity via different routes of exposure (oral, skin, inhalation) as displayed in product safety summaries (UBE Industries, Japan); it shows no marked health hazard properties and its acute toxicity is concluded to be very low (http://icca.cefic.org/Portal/SafetySummarySheets/634804519403177530_1,6‐hexanediol.pdf). The fact that the amphiphilic 1,6‐HD renders NPCs permeable to large nanoparticles without dissociation of barrier‐forming FG‐Nups leads us to postulate the following principles of action: (a) It interferes with the hydrophobic interactions between the FG‐Nups.^14^ This increases the physical gaps between the FG‐Nups and allows for the diffusion of larger molecules. (b) It collapses the extended, flexible and highly disordered brushes of FG‐Nups which form the entropic barrier as described in the “polymer brush” model.^21^ Larger molecules are generally unable to diffuse in this model as they cause a strain on the system by restricting the conformational freedom of the highly disordered FG‐Nups anchored to the scaffold due to their large molecular mass or volume.^22^ Disrupting the repulsion forces between the FG‐Nups (responsible for the generation of the entropic barrier) would thus lead to wider openings in the entropic barrier, which allow for the diffusion of larger molecules.

MI is able to facilitate the nuclear uptake of 70 kDa dextrans as we found out in our screening tests for selection of NNBs, but it fails to do so for 150 kDa pDNA, while 1,6‐HD manages to facilitate the nuclear uptake of both. This is likely due to the differences in the chemical structures of the two molecules. MI is quite hydrophilic due to its six hydroxyl groups. 1,6‐HD is less hydrophilic, as it has two hydroxyl groups bound to Carbons 1 and 6, whereas all Carbons from 2 to 5 are bound to hydrogen, granting it both a hydrophilic and a hydrophobic nature, each on a different side of the molecule. Due to this more balanced amphiphilic nature, 1,6‐HD might be able to interact more with the FG‐Nups, which have been shown to possess both hydrophobic and hydrophilic characteristics. The spacers in FG‐Nups are hydrophilic whereas the FG repeats themselves are hydrophobic.^2^ Owing to this fact, 1,6‐HD is able to target both the FG repeats through its hydrogen‐bound Carbons 2–5 and the spacers through its hydroxyl group‐bound Carbons 1 and 6. MI, on the other hand might only be able to target the hydrophilic spacers, which might not be enough to open the physical barrier wide enough for 150 kDa pDNA to go through.

## 5 CONCLUSIONS

Several studies show that amphiphilic alcohols render NPCs permeable to large macromolecules, using 70 kDa as an example. The studies are performed on yeast cells, except for very few utilizing human cell lines.^13^, ^16^ In a single study to date, 1,2‐TCHD is observed to enable nuclear delivery of single pDNA particles, while leading to fairly high cell mortality rates.^13^ This work introduces 1,6‐HD as a potent NBB, which facilitates passive delivery of pDNA into nuclei of human cells at rates ranging from 10% to 20%.

Two percentage of 1,6‐HD is sufficient to enable nuclear uptake of individual pDNA particles while 4% leads to a significant uptake. In light of this efficacy and the rather insignificant cytotoxicity even at fairly high concentrations, 1,6‐HD may serve as a promising lead substance for more specific NBBs for nanomedical applications.

## CONFLICT OF INTERESTS

The authors declare no conflicts of interest.
